# Not Only COVID-19: Prevalence and Management of Latent Mycobacterium Tuberculosis Infection in Three Penitentiary Facilities in Southern Italy

**DOI:** 10.3390/healthcare10020386

**Published:** 2022-02-18

**Authors:** Carmine Izzo, Annunziata Monica, Giuseppe De Matteis, Sebastiana De Biasi, Anna De Chiara, Antonio Maria Pagano, Eleonora Mezzetti, Fabio Del Duca, Alice Chiara Manetti, Raffaele La Russa, Marco Di Paolo, Aniello Maiese

**Affiliations:** 1 ASL SA, Dipartimento delle Attività Territoriali, U.O.S.D. Tutela Salute Adulti e Minori Area Penale, 84124 Salerno, Italy; carmine.izzo93@gmail.com (C.I.); annunziata.monica@gmail.com (A.M.); info@solentovacanze.it (G.D.M.); debiasisebastiana@gmail.com (S.D.B.); anna_de_chiara@libero.it (A.D.C.); a.pagano@aslsalerno.it (A.M.P.); 2 Department of Medicine, Surgery and Dentistry “Scuola Medica Salernitana”, University of Salerno, 84081 Baronissi, Italy; 3 Department of Surgical, Medical, and Molecular Pathology and Critical Care Medicine, University of Pisa, 56126 Pisa, Italy; eleonora.mezzetti@gmail.com (E.M.); a.manetti3@studenti.unipi.it (A.C.M.); marco.dipaolo@unipi.it (M.D.P.); 4 Department of Anatomical, Histological, Forensic and Orthopaedic Sciences, Sapienza University of Rome, 00185 Roma, Italy; fabio.delduca@uniroma1.it; 5 Department of Clinical and Experimental Medicine, University of Foggia, 71122 Foggia, Italy; raffaele.larussa@unifg.it

**Keywords:** prisons, tuberculin test, QuantiFERON test, latent tuberculosis

## Abstract

Latent Mycobacterium tuberculosis infection (LTBI) and active tuberculosis in prisoners are higher than the general population and are two public health concerns, especially in low- and middle-income countries. We conducted a cross-sectional study to determine the prevalence and the factors associated with LTBI among the inmate population detained in three Southern Italian penitentiaries. Tuberculin intradermal reaction skin test was performed on the inmates who agreed to participate in the study. In case of positivity, the QuantiFERON-TB test was performed. In those positive to QuantiFERON, chest X-ray films were performed, and treatment initiated. A total of 381 inmates accepted to participate. The prevalence of LTBI was 4.2%. In the analysis, LTBI was associated with no self-reported contact with active tuberculosis patients within the prisons, and 10% of subjects admitted the use of inhaled drugs. No HIV coinfections were found. No cases of active symptomatic tuberculosis were identified during the study period. Our results confirm that incarceration increases the risk of tuberculous infection. Non-EU nationality and a history of drug addiction appear to be major risk factors for tuberculosis infection in the penitentiary setting. Reinforcing tuberculosis control is essential to prevent its transmission in prisons.

## 1. Introduction

Tuberculosis (TB) is an infectious disease caused by various strains of mycobacteria, especially Mycobacterium tuberculosis, also called Koch’s bacillus [1]. Until the mid-twentieth century, TB was considered a serious disease, with high rates of morbidity and mortality. TB has now become more easily diagnosed and treatable in Western countries. This disease usually attacks the lungs (pulmonary TB) but can also affect other parts of the body (extrapulmonary TB). It is transmitted by droplets of saliva emitted with a dry cough [2]. Most infections turn out to be asymptomatic, meaning there is a latent infection. Latent tuberculosis infection (LTBI) could progress to active disease in 1 out of 10 cases and, if untreated, death has been reported in more than 50% of cases [3]. The classic symptoms are a chronic cough with blood-streaked sputum, night sweats, and weight loss, while a high fever is rare. Other organs can be affected by the infection with a consequent wide range of symptoms. Diagnosis is based on radiological examination (commonly a chest X-ray), a tuberculin skin test (TST), blood tests, microscopic examination, and microbiological culture of body fluids. Treatment may have compliance issues as it requires following a therapeutic regimen based on three anti-mycobacterial agents or more at a time. Antibiotic resistance is a pressing problem in coping with the disease. Prevention is based on screening and vaccination programs with the Bacillus Calmette–Guérin (BCG) vaccine [4,5,6]. TB in the prison population is a major public health concern, especially in low- and middle-income countries [7]. Regardless of the country’s economic status and burden of TB, the estimated prevalence of LTBI and active TB in prison is higher than that of the general population [1,2,8]. Italy is not an exception; studies of the incidence and prevalence of TB in prisons have reported values higher than those found in the general population [9,10]. TB in prisons remains poorly reported. For the 15 EU countries that reported data in 2019, the notification rate was 155 new TB recurrence cases per 100,000 inmates, which is a relatively high risk (11.4%) when compared to the general population in the same countries [11]. We wish to continue our efforts in screening and management of various diseases in the penitentiary facilities of the Salerno province [12,13,14,15,16]. For these reasons, the aim of the present study is to estimate the prevalence of LTBI in the prison population of Salerno, Vallo della Lucania, and Eboli penitentiary facilities in Southern Italy, and to identify the risk factors associated with LTBI.

TB happens to be a major public health problem also in the community population which cannot be controlled unless effective measures are taken to fight this disease in prisons [17,18,19,20]. In fact, TB is not limited to prisoners only, since it also affects the community and they interact with family members and prison staff during and after the period of their conviction [21,22,23,24]. “Stop TB”, set by the WHO, is the main global strategy for TB control [25]. This program highlights the need to promote TB control activities targeting prisoners because such individuals are at a high risk of latent infection with Mycobacterium tuberculosis and potentially developing the tuberculous disease. Even if the control and prevention of coronavirus disease 2019 (COVID-19) is currently a priority, we must keep in mind that TB is a disease with an again-increasing incidence, and it also represents a major public health issue in the prison setting [26,27,28]. One of the causes is the confinement context of overcrowding, where even the most elementary rules of preventive medicine cannot be enforced [29]. Other causes of the recurrence of the disease in prison include the high number of non-EU inmates, the high prevalence of HIV-positive inmates, the high incidence of substance abusers, and the lack of a suitable surveillance system [30,31]. By coughing, sneezing, or simply talking to each other, the bacteria enclosed in droplets are expelled into the closed environment of detention rooms and tend to float and remain in the air. These infected droplets can be easily inhaled by other inmates causing contagion [32,33].

The Salerno prison institute houses about 390 inmates. The Salerno institute consists of a women’s section (in a separate building), a section with “common” inmates, a section with “high security” inmates, and finally a section that houses inmates with problems of “prisoner-on-prisoner physical violence” and the “worker” detainees. In the institute, there is also a managed psychiatric unit. The Eboli prison institute houses about 50 inmates, with reduced custody for drug addiction treatment. Finally, the prison of Vallo della Lucania is characterized for hosting about 40 “sex offenders” inmates.

## 2. Materials and Methods

An observational cross-sectional study was conducted in prison facilities of the Salerno province to evaluate the prevalence of LBTI sustained by Mycobacterium tuberculosis. The Department of Territorial Activities, U.O.S.D. Protection for Adults and Minors of the ASL Salerno Criminal Area, operates in three different penitentiary facilities: Eboli, Vallo della Lucania, and Salerno. In June 2020, the Mantoux TST was performed on inmates of these three different penitentiary institutes who gave consent. Informed consent was executed privately one inmate at a time in written form upon scheduled medical visits in the prison sections, by the prison doctor in duty. No incentives were offered to the inmate participants.

The data processing complied with the general authorization for scientific research purposes granted by the Italian Data Protection Authority (1 March 2012 as published in Italy’s Official Journal no. 72 dated 26 March 2012) since the data do not entail any significant personalized impact on the data subjects. Approval by an institutional and/or licensing committee was not required since experimental protocols were not applied in the study.

Protocols and screening were conducted as suggested by the World Health Organization and in conformity with the ethical guidelines of the 1975 Declaration of Helsinki.

One prison section at the time was tested. The eligibility criteria were as follows: who freely accepted to participate; having had no previous treatment for tuberculosis; having no tuberculosis disease at the study outset. The following variables were also evaluated: sex, weight, height, comorbidities such as cancer, transplants, diabetes mellitus (DM), hypertension (HT) rheumatoid arthritis (RA), chronic obstructive pulmonary disease (COPD), chronic kidney disease (CKD), psychiatric disorders, and any other immunosuppressive disease, abuse of drugs (inhaled, injected, or smoked) or alcohol, along with the quantity and time of consumption, fever, weight loss, night sweats, hemoptysis, and BCG vaccination scar.

Variables were collected from prison records as at inmate entrance, clinical visits are carried out in the central infirmaries of the prisons. During the visits, anamnestic and clinical information are privately collected by the doctor on duty. The reliability of inmate declarations is a limitation that could lead to recall biases.

The statistical data analysis was expressed as frequencies, percentages, and Fisher’s test. All statistical analyses were performed using the Statistical Program for Social Sciences (SPSS^®^) v. 20 for MacIntosh^®^ (IBM Corp., Armonk, NY, USA).

The “TUBERTEST^®^” was used as indicated in the package leaflet that it recommends in 1 bottle of 10 doses of 1 mL, imported by SANOFI S.p.A. The STS consisted of subcutaneous administration of 0.1 mL (2 tuberculin units) of M. tuberculosis PPD RT23 (State Serum Institute, Copenhagen, Denmark) on the left forearm. TSTs were read 72 h after being administered, by measuring the maximum transverse diameter of induration with a millimetre ruler. The LTBI status was determined by the size of TST induration in the absence of a reported previous positive test. An induration of ≥10 mm was considered a positive result for HIV-negative individuals, and an induration of ≥5 mm was considered a positive result for HIV-infected individuals.

All subjects with positive Mantoux performed blood sampling for QuantiFERON-TB. Subsequently, the protocol provided that positive subjects took chest X-rays films. In case of positivity to X-rays or onset of symptoms, they would be hospitalized, while if negative they would start therapy for LTBI. We followed the Italian guidelines, which indicate to administer isoniazid and rifampin for three months or isoniazid for six months [34]. Therapy compliance and adverse effects were eventually evaluated.

## 3. Results

The general inmate population at the time of the screening was composed of 420 prisoners eligible to participate in the study, 383 (91.19%) men, 37 (8.81%) women, and 83 (19.76%) foreigners. Among these 420, the prevalence of comorbidities was: 6 inmates with COPD, 1 inmate with CKD, 37 inmates with HT, 18 inmates with type 2 DM, 80 with anxious-depressive syndrome, and one HIV (human immunodeficiency virus)-positive. Age ranges were as follows: 23 inmates between 60–80 years (of these, 1 refused); 61 inmates between 50–69 years (of these nine refused); 265 inmates between 30–49 years (29 refused); 71 inmates between 18–29 years (none refused). Overall, 39 (9.29%) inmates refused to be screened, two (5.13%) of them were female. Education level was homogeneous as 418 inmates attended compulsory school and two graduates (neither were positive). The mean incarceration time was 52 months. Prisoners who accepted to participate in the study were 381 (90.71%) and of these 330 (78.1%) were Italian, 51 (21.9%) were foreigners, 110 (28.87%) were people who inject drugs (PWIDs). There was only one HIV-positive inmate; he accepted to be tested and resulted negative to the “TUBERTEST^®^”. Table 1 shows the main characteristics of the inmate population and of our sample.

Among the 381 participants, 31 (8.13%) resulted positive to the “TUBERTEST^®^”. Further information about the characteristics of these subjects is provided in Table 2. As protocol, to those who agreed, blood sampling for QuantiFERON-TB was acquired and 18 (4.72%) inmates resulted positive. Among these 18 positives, 10 were Italian, 8 were foreigners, 1 was female, and 5 were PWIDs. The prevalence of comorbidities in the positive inmates was the following: one inmate with COPD, three with HT, and two with type 2 DM.

These patients were put in sanitary isolation and brought to the nearest hospital for chest X-rays. Since none were symptomatic, hospitalization was not required.

**Figure 1:**
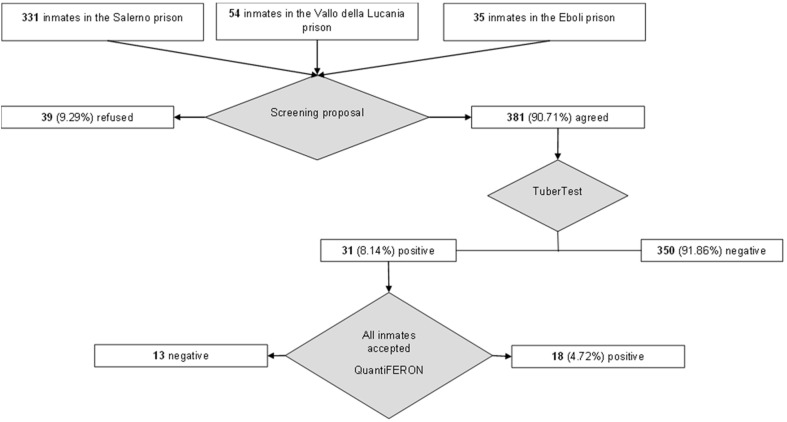
A flowchart of our inmates’ tuberculosis screening protocol.

Table 3 shows the frequency of each variable among the LTBI-positive and LTBI-negative groups. Figure 1 shows the flowchart of our tuberculosis screening protocol. Table 4 shows the results of the Fisher’s test.

According to guidelines, latent TB therapy was initiated, and fortunately, only one inmate refused. Overall, only seven inmates completed the treatment in our penitentiary facilities due to six transfers to other prisons and four releases from prison. Compliance of these ten subjects was unfortunately not evaluable since we could not perform a follow-up. On the other hand, of the seven treated inmates, none reported side effects to the TB treatment and/or onset of TB symptoms during the following 6 months.

## 4. Discussion

According to the World Health Organization (WHO), about two billion people, or one-third of the world population, have been exposed to the tuberculosis pathogen [15]. Annually, 8 million people become ill with tuberculosis, and 2 million patients die from the disease worldwide [16]. TB prevention and control have two parallel approaches. On one hand, people with TB and those close to them are identified and treated. Identifying infections often involves examining high-risk groups for TB, such as prison inmates. On the other hand, children are vaccinated against TB. Unfortunately, no vaccine available guarantees reliable protection for adults [17]. The WHO “Global tuberculosis report 2020” confirms that tuberculosis is among the top ten causes of death in the world [18]. It is estimated that in 2019, 10 million people received a new diagnosis of TB (incident cases), 88% of whom were adults (age above 15 years), and 8.2% resulted positive for human immunodeficiency virus (HIV) co-infection [11,19,20,21]. The most recent data relating to Italy were published in the joint document ECDC and WHO Europe “Tuberculosis surveillance and monitoring in Europe 2021 (2019 data)” and confirm that Italy is one of the countries with the lowest incidence of the disease (less than 20/100,000) [21,22]. In 2019, 3346 cases of tuberculosis were notified; this corresponds to an incidence in the population of 5.5/100,000 inhabitants, a slight decrease compared to the previous year (3912 cases with an incidence of 6.5/100,000). From 2015 to 2019, the notification rate of TB decreased on average by 2.8% per year. Of the total reported cases, 56.2% occurred in patients of foreign origin [21].

The difficulties encountered in prison for the control of TB include the lack of possibility, in some Penitentiary Health Centers, to organize the execution of Mantoux intradermal reaction to newly arrived inmates, the need to send the positive prisoners to external facilities with the penitentiary police escort to perform a chest X-ray, and the difficulty in guaranteeing sanitary isolation with all the necessary precautions while still guaranteeing air for daily walking [35].

The magnitude of LTBI within the Italian prison system, as previously mentioned, remains mostly unknown. The prevalence observed in our study is 4.72%. We found that being foreign is related with a higher frequency of LTBI (*p* value < 0.01). Overall, 8 out of 51 foreign prisoners (15.69%) were positive. Surprisingly, we did not find a significative correlation between LTBI and the use of injected drugs. Treatment management was difficult due to the long period of therapy required that clashes with the prison reality. In fact, only seven (38.88%) inmates completed the 6 months therapy in our penitentiary facilities. The lack of therapeutic compliance derived from one refusal, six inmates transfer to other penitentiary facilities, and four releases from prison. We advocated for the conclusion of the treatment to both the inmates and the new hosting penitentiary facility. Unfortunately, we were not able to have feedback on the therapy follow-through, and this is a limit of our study. Another limit is that we considered only three facilities. There is no clear way to predict the development of tuberculosis among prisoners, especially if asymptomatic. In a resource-poor setting such as the prison environment, only screening can be a viable way [36,37]. More data are needed to implement our knowledge on risk factors of LTBI among prisoners to create adequate screening programs and preventive strategies. Up to now, a possible screening protocol could be the one we adopted. In particular, we suggest that all new prison inmates should undergo a TST to select those who require further diagnostic investigations and treatment. TST is a low-cost exam compared to the therapy costs in case of TB spreading among prisoners. The complete follow-up of all the 17 latent TB infected inmates that agreed to the therapy would have allowed us to evaluate the efficacy of our protocol in containing TB in our penitentiary facilities. Unfortunately, this was not possible and is another limitation of our study.

Recent literature reviews show a risk of false-positive results in tuberculin tests in subjects who received the BCG vaccination. If BCG vaccination is given after the first year of life, as presumably in our cases, more frequent, persistent, and larger skin test reactions are produced [38]. Tuberculin skin test results can be altered from 10 up to 55 years after vaccination. Otherwise, exposure to non-tuberculous mycobacteria (NTM) is not a significant cause of a false-positive, except in populations with a high prevalence of NTM sensitization and a very low prevalence of TB infection [39]. In brief, another limitation of our study is that the lack of anamnestic data did not allow us from identifying the vaccinated subjects and therefore possible false-positive cases.

## 5. Conclusions

In conclusion, our results confirm that incarceration increases the risk of tuberculous infection. According to our data, the high number of non-EU prisoners seems to be one of the main factors related to TB infection recurrence in prisons. Reinforcing TB control is essential to prevent its transmission in penitentiary facilities. To develop proper screening and preventive programs, it is fundamental to highlight the factors that increase the risk of LTBI in this kind of population. Therefore, more studies are needed in this field. According to the current knowledge, we propose to perform a TST on all prisoners, especially to the new inmates, to select those needing further investigations and then TB therapy. Only an adequate screening strategy could help contain the again-increasing incidence of LTBI in prisons.

## Figures and Tables

**Table 1:** Characteristics of the study population (*n* = 420) and of the prisoners who accepted the TuberTest (*n* = 381). ADS indicates anxious-depressive syndrome; COPD, chronic obstructive pulmonary disease; CKD, chronic kidney disease; DM2, type 2 diabetes mellitus; HIV, human immunodeficiency virus; HT, hypertension, PWIDs, people who inject drugs.

Subject Characteristics		Overall (*n* = 420)	Testing Accepted (*n* = 381)
Sex	Male	383	346
	Female	37	35
Nationality	Italian	337	330
	Not Italian	83	51
Age	60–80 years	23	22
	50–59 years	61	52
	30–49 years	265	236
	18–29 years	71	71
Comorbidities	COPD	6	6
	CKD	1	0
	HT	37	36
	DM2	18	18
	ADS	80	75
	HIV+	1	1
Education level	Compulsory school	418	379
	Graduation	2	2
PWIDs	Yes	119	110
	No	301	271

**Table 2:** Frequency of each variable among our cases considering the results of the TuberTest and QuantiFERON. ADS indicates anxious-depressive syndrome; COPD, chronic obstructive pulmonary disease; CKD, chronic kidney disease; DM2, type 2 diabetes mellitus; HIV, human immunodeficiency virus; HT, hypertension; PWIDs, people who inject drugs.

Subject Characteristics		Total	TuberTest Positive	TuberTest Negative	QuantiFERON Positive	QuantiFERON Negative
Total		381	31	350	18	13
Sex	Male	346	30	316	17	13
	Female	35	1	34	1	0
Nationality	Italian	330	17	313	10	7
	Not Italian	51	14	37	8	6
Age	60–80 years	22	10	12	7	3
	50–59 years	52	11	41	6	5
	30–49 years	236	5	231	3	2
	18–29 years	71	5	66	2	3
Comorbidities	COPD	6	3	3	1	2
	CKD	0	-	-	-	-
	HT	36	15	21	3	12
	DM2	18	8	10	2	8
	ADS	75	7	62	0	7
	HIV+	1	0	1	-	-
Education level	Compulsory school	379	31	348	18	13
	Graduation	2	0	2	-	-
PWIDs	Yes	110	11	99	5	6
	No	271	20	251	13	7

**Table 3:** Frequency of each variable among the LTBI-positive group and LTBI-negative group. ADS indicates anxious-depressive syndrome; COPD, chronic obstructive pulmonary disease; DM2, type 2 diabetes mellitus; HIV, human immunodeficiency virus; HT, hypertension; LTBI, latent tuberculosis infection; PWIDs, people who inject drugs.

Subject Characteristics		Total	LTBI	No LTBI
Total		381	18 (4.72%)	363 (95.28%)
Sex	Male	346	17 (4.91%)	329 (95.09%)
	Female	35	1 (2.86%)	34 (97.14%)
Nationality	Italian	330	10 (3.03%)	320 (96.97%)
	Not Italian	51	8 (15.69%)	43 (84.31%)
Age	60–80 years	22	7 (31.82%)	15 (68.18%)
	50–59 years	52	6 (11.54%)	46 (88.46%)
	30–49 years	236	3 (1.27%)	233 (98.73%)
	18–29 years	71	2 (2.82%)	69 (97.18%)
Comorbidities	COPD	6	1 (16.67%)	5 (83.33%)
	HT	36	3 (8.33%)	33 (91.67%)
	DM2	18	2 (11.11%)	16 (88.89%)
	ADS	75	0	75 (100%)
	HIV+	1	0	1 (100%)
Education level	Compulsory school	379	18 (4.75%)	361 (95.25%)
	Graduation	2	0	2 (100%)
PWIDs	Yes	110	5 (4.54%)	105 (95.45%)
	No	271	13 (4.8%)	258 (95.2%)

**Table 4:** The data were statistically analyzed. Fisher’s exact test was performed (*p* value < 0.05). The test showed a significant statistical correlation between LTBI and the variables age (age older than 49 years) and nationality (not Italian people). The low frequency of comorbidities among the LTBI-positive group, as well as the fact that all the positive subjects attended only compulsory school, did not allow performing further analysis on these variables. LTBI indicates latent tuberculosis infection; PWIDs, people who inject drugs.

Subject Characteristics		LTBI (*n* = 18)	No LTBI (*n* = 363)	Total (*n* = 381)	Fisher’s Test *p* Value (*p* < 0.01)
Sex	Male	17 (4.91%)	329 (95.09%)	346	1
	Female	1 (2.86%)	34 (97.14%)	35	
Nationality	Not Italian	8 (15.69%)	43 (84.31%)	51	0.0009
	Italian	10 (3.03%)	320 (96.97%)	330	
Age	>49 year-old	13 (17.57%)	61 (82.43%)	74	0.00001
	≤49 year-old	5 (1.63%)	302 (98.37%)	307	
PWIDs	Yes	5 (4.54%)	105 (95.45%)	110	1
	No	13 (4.8%)	258 (95.2%)	271	

## Data Availability

Not applicable.
